# Factors associated with quality of life in patients with depression: A nationwide population-based study

**DOI:** 10.1371/journal.pone.0219455

**Published:** 2019-07-11

**Authors:** Yunji Cho, Joo Kyung Lee, Do-Hoon Kim, Joo-Hyun Park, Moonyoung Choi, Hyun-Jin Kim, Myung-Ji Nam, Kang-Uk Lee, Kyungdo Han, Yong-Gyu Park

**Affiliations:** 1 Department of Psychiatry, Samsung Medical Center, Sungkyunkwan University, College of Medicine, Seoul, Republic of Korea; 2 Department of Internal Medicine, Korea University Guro Hospital, Korea University College of Medicine, Seoul, Republic of Korea; 3 Department of Family Medicine, Korea University Ansan Hospital, Korea University College of Medicine, Ansan-si, Republic of Korea; 4 Department of Biostatics, Catholic University College of Medicine, Seoul, Republic of Korea; Harvard University, UNITED STATES

## Abstract

**Background:**

Depression, one of the most costly and common mental disorders, is reported to be associated with lower quality of life (QoL) in several studies. Improved understanding of the associated factors with QoL is necessary to optimize long-term outcomes and reduce disability in patients with depression. Therefore, the aim of this study was to identify factors that are associated with lower QoL among patients with depression.

**Methods:**

The study was based on the Korea National Health and Nutrition Examination Survey, a cross-sectional health examination, years 2008 to 2014. The final analyzed sample consisted of a total of 1,502 study subjects who had been diagnosed by clinicians as having depression. A multivariate logistic regression model was performed to exam the association between the clinical characteristics (age, sex, demographic and health-related characteristics) and QoL. Analysis of covariance was also used to analyze EQ-5D according to mental health.

**Results:**

Older age, lower level of education, lower income, worse subjective perception of health, unemployment, obesity and mental health struggles were found to be significantly associated with low QoL in depressive individuals after adjustment for multiple covariates.

**Conclusions:**

This study has outlined grounding data in identifying patients who are at risk of QoL impairment. Policy makers should direct their interests to these individuals and provide appropriate management.

## Introduction

As trends in major health problems shift from acute infectious diseases to chronic diseases, the concept of health is moving toward emphasizing not only an increase in life expectancy but also quality of life (QoL). The individual perception of health status is represented by the concept of health-related QoL.[1]

Depression is recognized as one of the most costly and common disorders worldwide, and its large economic burden is derived from its high prevalence and substantial functional disabilities entailed in the illness. [2] Functional impairment inevitably leads to deteriorations in QoL, or subjective perception of well-being in social, occupational, or health-related dimensions. [3–6] Several studies have explored and confirmed the association between depression and QoL. Depression has been found to contribute to the development of several chronic medical diseases, including heart disease and diabetes, resulting in further disability and low QoL. [5–8] Meta-analyses have been conducted in examining whether pharmacotherapy and/or psychotherapy are effective in enhancing QoL via improving depressive symptoms, and have found mixed results. [4] Public concerns surrounding depression have been growing, regarding its widespread prevalence and substantial impact on public health. [9, 10]

Thus, in treating depression, the optimal treatment outcome has been recognized as full remission of depressive symptoms and improvements in psychosocial functioning. Partial remission and residual depressive symptoms have been associated with impaired quality of life, and subsequently, burden in healthcare and social welfare. [11] QoL was reported to be associated with improved adherence and response to treatment in patients with depression. [12, 13] Health-related QoL (HRQoL) has been known to be an independent factor affecting various medical outcomes, such as death or re-admission, and is an important consideration for health care interventions.[14]

Improving QoL is an essential goal in optimizing long-term outcomes and reducing disabilities in patients with depression. Understanding the demographics and health-related factors of associated individuals provides clinicians and policy makers with a clinical overview, and helps provide a guideline in establishing a long-term management plan for depression. The economic and social burden of depression are reaching substantial size, and by locating which individuals are at higher risk, the limited clinical resources can be allocated with higher efficiency and effectivness.

Therefore the present study aimed to identify associated factors with QoL of patients with depression, based on data from the Korea National Health and Nutrition Examination Survey (KNHANES) 2008 to 2014. To analyze the independent association between sociodemographic and clinical factors and QoL in patients with depression, we included the following various variables: age, sex, residential area, education, household income, spouse, employment, current smoking, drinking, regular exercise, obesity, metabolic syndrome, subjective perception of health and mental health status.

## Materials and methods

### Study population and data collection

This study was based on the Korea National Health and Nutrition Examination Survey (KNHANES), a cross-sectional health examination and survey conducted by Ministry of Health and Welfare, Korea Centers for Disease Control and Prevention, and Division of Health and Nutrition Survey. KNHANES was launched in 1998 in an effort to establish a nationwide surveillance system that monitors the health and nutritional status of the Korean population. Data are collected year-round and assessed annually through a rolling sampling survey method. The target population is noninstitutionalized South Korean citizens sampled following a stratified multi-stage clustered probability design. Every year, 192–200 primary sampling units (PSUs) are drawn randomly from approximately 200,000 geographically-defined PSUs, and 20–23 households are then sampled from each PSU. A health examination, health interview, and nutrition survey are then conducted after participants sign informed consent forms.

The data used in the present study were retrieved from the data for the years 2008 to 2014, during which 58,306 individuals aged > 1 year were sampled (9,308 in 2008; 10,078 in 2009; 8,473 in 2010; 8,055 in 2011; 7,645 in 2012; 7,580 in 2013; 7,167 in 2014). Those aged less than 19 years were excluded, as they were not surveyed with the EQ-5D and EQ-VAS. Depression was defined based on study subjects’ self- administered reports of having been diagnosed with depression by clinicians. After excluding incomplete data, a total of 1,502 study subjects who had been diagnosed with depression were analyzed in the present study.

### Assessment of QoL and mental health

Data regarding QoL and mental health were obtained by means of a questionnaire, aided by trained supervisors. Measurement of health-related QoL was based on evaluations established by EuroQol in participants of age ≥ 19 [15]. EuroQol consists of a health-status descriptive system (EQ-5D) and a visual analogue scale (EQ-VAS). EQ-5D is a standardized instrument that comprises of five dimensions: mobility, self-care, usual activities, pain/discomfort, and anxiety/depression. Each dimension has three levels (EQ-5D-3L): no problems, some problems, and extreme problems. Respondents self-report their perceived level of performance in accordance with the survey items. Responses for all five dimensions are merged into a single index score using a valuation set developed by the Korean Centers for Disease Control and Prevention [16]. Scores range from −0.171 to 1. A score of 1 indicates no problems in all dimensions, 0 implies death, and negative values describe a health condition worse than death. The EQ-VAS records respondents’ self-rated health on a scale ranging from 0 (worst imaginable health state) to 100 (best imaginable health) [15].

Mental health was assessed in adults ≥ 19 years of age based on responses to a self-administered questionnaire. Subjects were questioned about the following items: mental stress, melancholia, suicidal ideation, experience of consulting professionals, and suicide attempts. Melancholia was assessed by a “yes” or “no” answer to the following question: “Have you felt sadness or despair that affects your daily life for more than 2 weeks over the past year?”[17] The experience of consulting professionals was assessed by a “yes” or “no” to the following questions; “Have you visited any healthcare institutions, or received consultation through the Internet, telephone, etc. regarding your mental health problems during the past year?” Suicidal ideation was assessed by the question “In the last 12 months, did you think about committing suicide?” A “yes” or “no” response was also used to determine whether the subjects had suicidal thoughts; if the subject answered “yes,” they were asked about their suicide attempts, if any. This indicator is a well-documented predictor of suicide attempts that has been previously used in other surveys of adults [18] and in previous KNHANES studies. In addition, participants reported their level of stress as none, mild, moderate, or severe.

### Statistical analyses

Socio-demographic and clinical characteristics are presented as mean ± standard error (SE) or as % (SE). Correlation analyses were utilized to assess and visualize relation between age/sex and each item of the EQ-5D. Odds ratios (ORs) for the five EQ-5D dimensions were calculated for various sample characteristics. A multivariate logistic regression model was used to adjust the clinical characteristics (age/sex, demographic, and health-related characteristics) and assess whether statistical significance was sustained. Thus, the inclusion of each index as an explanatory variable depends on the dependent variable. Variables with *p* < 0.1 in the univariate test were selected as candidates for the multivariate model. Finally, analysis of covariance (ANCOVA) was performed on the EQ-5D by adjusting a variety of clinical characteristics, and *p*-values of less than 0.05 were considered statistically significant. Statistical analyses were performed using SAS 9.2 software (SAS institute, Inc., Cary, NC).

## Results

Table 1 displays the sociodemographic characteristics and health behaviors of the study sample in relation to QoL as expressed in the EQ-5D and EQ-VAS. Both the EQ-5D and EQ-VAS showed lower scores for those aged ≥ 70 than those aged 60–69, indicating lower perceived QoL in older age groups. Sex, place of residence, and smoking did not show definite statistical correlation in terms of QoL. Drinkers had higher EQ-5D scores and those who regularly exercise had higher EQ-5D and EQ-VAS scores. Higher EQ-5D and EQ-VAS scores were noted in subjects with higher socioeconomic status. Education level, income, and marital and current employment status were positively correlated with QoL. Physical health showed significant differences with respect to QoL. Patients with worse subjective perception of health had lower EQ-5D and EQ-VAS. Subjects with body mass index (BMI) ≥ 25 or central obesity (weight circumference ≥ 90 cm for men and ≥ 85 cm for women) had lower EQ-5D scores. Likewise, patients with comorbidities such as metabolic syndrome, hypertension, diabetes, and hypercholesterolemia showed lower QoL. In terms of mental health, an experience of substantial mental stress, depressive mood, clinical consultation, suicidal ideation, and suicide attempts all led to significant decreases in EQ-5D and EQ-VAS. Among these, depressive mood and suicidal ideation led to difficulties in every dimension of EQ-5D (mobility, self-care, usual activities, pain/discomfort, and anxiety/depression).

**Table 1:** Socio-demographic characteristics and health behaviors of the study subjects.

	Frequency % (SE)	Mobility	Self-care	Usual activity	Pain /discomfort	Anxiety /depression	EQ-5D	EQ_VAS
**Age (years)**								
19–59	29.6(1.6)	11.4(2.3)	3.3(1.4)	10.3(2.2)	34.3(3.4)	39.1(3.3)	0.915±0.011	64.897±1.444
60–69	51.4(1.6)	28.3(2)	6.5(1.1)	21.6(1.7)	42.3(2.2)	39.8(2.2)	0.868±0.007	66.18±0.906
≥70	19(1)	56.3(2.7)	23.4(2.5)	43(2.8)	61(2.6)	44.3(2.8)	0.766±0.012	60.89±1.291
*p*-value*		< 0.001	< 0.001	< 0.001	< 0.001	0.455	< 0.001	0.003
**SEX**								
Male	23.6(1.4)	26.6(3.3)	11.2(2.4)	25.5(3.3)	43.7(3.7)	35.9(3.6)	0.861±0.016	67.485±1.627
Female	76.4(1.4)	29.2(1.5)	8(0.8)	21.3(1.3)	43.4(1.7)	41.9(1.8)	0.863±0.005	64.064±0.721
*p*-value		0.472	0.168	0.221	0.953	0.133	0.931	0.061
**Place of residence**								
Rural	17.4(1.5)	33(3)	12.4(2)	25.7(3)	47.3(3.5)	37.6(3.7)	0.85±0.011	65.054±1.247
Urban	82.6(1.5)	27.7(1.6)	8(0.9)	21.6(1.4)	42.7(1.8)	41.1(1.8)	0.865±0.006	64.804±0.741
*p*-value		0.112	0.028	0.195	0.237	0.406	0.240	0.863
**Smoking**								
Non or Ex	82.3(1.4)	29.8(1.5)	8.7(0.9)	22(1.3)	43.4(1.7)	39.5(1.7)	0.866±0.005	65.391±0.709
Current smoker	17.7(1.4)	22.8(3.5)	9.1(2.6)	23.6(3.7)	43.7(4.2)	45(4.3)	0.846±0.02	62.367±1.829
*p*-value		0.082	0.885	0.697	0.947	0.217	0.360	0.132
**Drinking**								
No	56.2(1.6)	34.2(1.9)	10.2(1.1)	29(1.8)	47.7(2.1)	43.2(2.1)	0.841±0.007	64±0.815
Drinker	43.8(1.6)	21.7(2)	7.1(1.3)	13.9(1.6)	38.5(2.5)	36.9(2.5)	0.888±0.009	65.782±1.167
*p*-value		< 0.001	0.099	< 0.001	0.005	0.052	< 0.001	0.231
**Exercise**								
No	62.5(1.5)	31.2(1.8)	10.1(1.1)	23.5(1.6)	43.9(2.1)	40.5(2.1)	0.853±0.008	63.724±0.841
Regular exercise	37.5(1.5)	24.4(2.2)	6.5(1.1)	20.3(2)	43.1(2.5)	40.5(2.6)	0.878±0.007	66.632±0.96
*p*-value		0.020	0.028	0.234	0.815	0.990	0.016	0.020
**Education levels**								
Elementary to Middle (≤9yr)	45.9(1.6)	43.6(2.1)	13.7(1.5)	33.8(2)	53.1(2.1)	45.6(2.3)	0.809±0.008	62.496±0.961
High to University (≥10yr)	54.1(1.6)	15.9(1.7)	4.6(0.9)	12.5(1.5)	35.4(2.2)	36.1(2.2)	0.907±0.007	66.947±0.947
*p*-value		< 0.001	< 0.001	< 0.001	< 0.001	0.002	< 0.001	0.002
**Household Income**								
Others	74.9(1.4)	20.6(1.5)	4.9(0.6)	13.9(1.2)	39.1(1.9)	36.2(1.8)	0.897±0.004	66.592±0.759
Lowest quartile	25.1(1.4)	52.2(3)	19.4(2.5)	46.2(2.8)	56(2.9)	53.8(3.1)	0.762±0.015	59.387±1.5
*p*-value		< 0.001	< 0.001	< 0.001	< 0.001	< 0.001	< 0.001	< 0.001
**Spouse**								
No	29.7(1.5)	37.7(3)	13.6(2.1)	32.4(2.8)	52.5(3.2)	46.8(3.1)	0.814±0.012	61.894±1.199
Yes	70.3(1.5)	25.4(1.6)	7.1(0.9)	18.1(1.3)	40.3(1.9)	37.3(1.9)	0.882±0.005	66.279±0.776
*p*-value		< 0.001	< 0.001	< 0.001	< 0.001	0.008	< 0.001	0.002
**Self-perception of health**								
Good	15.3(1.1)	16.9(3)	3.6(1.7)	8.4(2.4)	23.1(3.4)	26.4(3.6)	0.929±0.008	80.631±1.004
Usually	41.5(1.6)	17.6(1.9)	4.1(0.9)	9.6(1.3)	29.8(2.4)	29.6(2.4)	0.92±0.005	67.676±1.018
Bad	43.2(1.6)	43.4(2.4)	15.1(1.6)	39.4(2.2)	63.9(2.4)	55.9(2.5)	0.784±0.01	56.695±0.927
*p*-value*		< 0.001	< 0.001	< 0.001	< 0.001	< 0.001	< 0.001	< 0.001
**Employment status**								
Yes	43.9(1.6)	19.1(1.7)	3.6(0.8)	13.1(1.4)	35.4(2.3)	37(2.4)	0.905±0.005	66.983±0.981
No	56.1(1.6)	36(2)	12.8(1.3)	29.5(1.9)	49.8(2.1)	43.2(2.1)	0.829±0.008	63.13±0.864
*p*-value		< 0.001	< 0.001	< 0.001	< 0.001	0.041	< 0.001	0.003
**Obesity**								
BMI < 25	65.7(1.6)	24.6(1.6)	7(0.8)	19.7(1.5)	39.1(1.9)	38.5(1.9)	0.88±0.005	65.226±0.837
BMI ≥ 25	34.3(1.6)	36.3(2.7)	12.2(1.9)	27.4(2.3)	51.8(2.8)	44.3(2.9)	0.828±0.012	64.106±1.068
*P* value		< 0.001	0.004	0.004	< 0.001	0.096	< 0.001	0.417
**Central obesity**								
No	56.8(1.7)	21.7(1.8)	6.5(1)	17.9(1.6)	37.8(2.2)	36.8(2.2)	0.891±0.006	65.47±0.935
Yes	43.2(1.7)	37.7(2.2)	11.8(1.5)	28.1(2)	51.1(2.4)	45.3(2.5)	0.825±0.01	64.095±0.933
*p*-value		< 0.001	0.002	< 0.001	< 0.001	0.011	< 0.001	0.309
**Perceived Stress**								
No	50.3(1.6)	28.5(1.9)	7.7(1.1)	19.7(1.7)	38.5(2.1)	26(2)	0.889±0.006	68.602±0.853
Yes	49.7(1.6)	28.7(2)	9.8(1.4)	25(2)	48.6(2.3)	55.1(2.4)	0.836±0.009	61.044±1.02
*p*-value		0.934	0.222	0.045	0.001	< 0.001	< 0.001	< 0.001
Melancholia								
No	56.1(1.6)	24.3(1.7)	6.6(0.9)	17.6(1.6)	38.5(2.2)	26.7(2)	0.898±0.005	68.265±0.828
Yes	43.9(1.6)	34.1(2.3)	11.5(1.5)	28.3(2)	49.9(2.3)	58.1(2.4)	0.817±0.01	60.473±1.03
*p*-value		0.001	0.003	< 0.001	0.000	< 0.001	< 0.001	< 0.001
**Suicidal ideation**								
No	57.2(1.5)	24.1(1.7)	5.9(0.9)	17.1(1.5)	37.4(2)	28.7(1.9)	0.9±0.005	69.023±0.762
Yes	42.8(1.5)	34.7(2.3)	12.7(1.5)	29.3(2.1)	51.6(2.5)	56.1(2.4)	0.812±0.01	60.099±1.099
*p*-value		0.000	< 0.001	< 0.001	< 0.001	< 0.001	< 0.001	< 0.001
**Professional consultation**								
No	75.5(1.4)	28.8(1.6)	8.6(0.9)	21(1.4)	43.9(1.8)	37.2(1.8)	0.869±0.006	65.6±0.7
Yes	24.5(1.4)	27.9(2.8)	9.3(1.7)	26.3(2.7)	42.2(3.2)	50.5(3.3)	0.843±0.012	62.8±1.3
*p*-value		0.774	0.700	0.059	0.647	0.000	0.045	0.057
**Suicide attempt**								
No	92.9(0.8)	27.9(1.4)	8(0.8)	20.7(1.3)	42.8(1.7)	38.6(1.7)	0.871±0.005	65.432±0.674
Yes	7.1(0.8)	38.1(5.9)	18.2(4.8)	42.8(6.1)	52.9(6.4)	64.1(6.1)	0.75±0.03	57.793±2.914
*p*-value		0.072	0.005	< 0.001	0.127	0.000	< 0.001	0.012
**Metabolic Syndrome**								
No	66.4(1.6)	21.1(1.7)	5.9(0.9)	17.5(1.6)	39.3(2.1)	35.9(2.1)	0.892±0.005	65.736±0.84
Yes	33.6(1.6)	41.1(2.8)	12.1(1.9)	29.8(2.4)	50(2.8)	46.2(2.8)	0.819±0.013	63.945±1.16
*p*-value		< 0.001	0.001	< 0.001	0.002	0.003	< 0.001	0.215
**Hypertension**								
No	70.1(1.4)	21.9(1.6)	6(0.8)	17.6(1.4)	38.7(2)	38.6(2)	0.888±0.005	65.983±0.783
Yes	29.9(1.4)	44.2(2.6)	15.3(2.1)	33.3(2.5)	54.7(2.5)	44.7(2.7)	0.803±0.013	62.189±1.295
*p*-value		< 0.001	< 0.001	< 0.001	< 0.001	0.063	< 0.001	0.015
**Diabetes**								
No	90.4(0.9)	25.8(1.5)	7.4(0.9)	20(1.4)	41.3(1.8)	38.3(1.8)	0.873±0.006	65.27±0.748
Yes	9.6(0.9)	42.8(4.7)	12.8(3.1)	35.2(4.5)	54.3(5)	47.6(5)	0.829±0.015	63.999±2.118
*p*-value		< 0.001	0.040	< 0.001	0.014	0.070	0.007	0.576
**Hyperlipidemia**								
No	82.7(1.3)	25(1.6)	7.4(0.9)	20.3(1.4)	40.2(1.9)	36.9(1.9)	0.882±0.005	65.671±0.739
Yes	17.3(1.3)	38.7(3.8)	10.3(2.6)	26.4(3.5)	53.3(3.9)	49.6(3.9)	0.806±0.02	62.881±2.076
*p*-value		< 0.001	0.242	0.083	0.002	0.003	< 0.001	0.210

Socio-demographic and clinical characteristics were presented as mean ± standard error (SE) or as % (SE).

*P-values were obtained by analysis of variance. Other all *p*-values for continuous variables or categorical variables were obtained by two-sample *t*-test or chi-square test, respectively.

**Fig 1:**
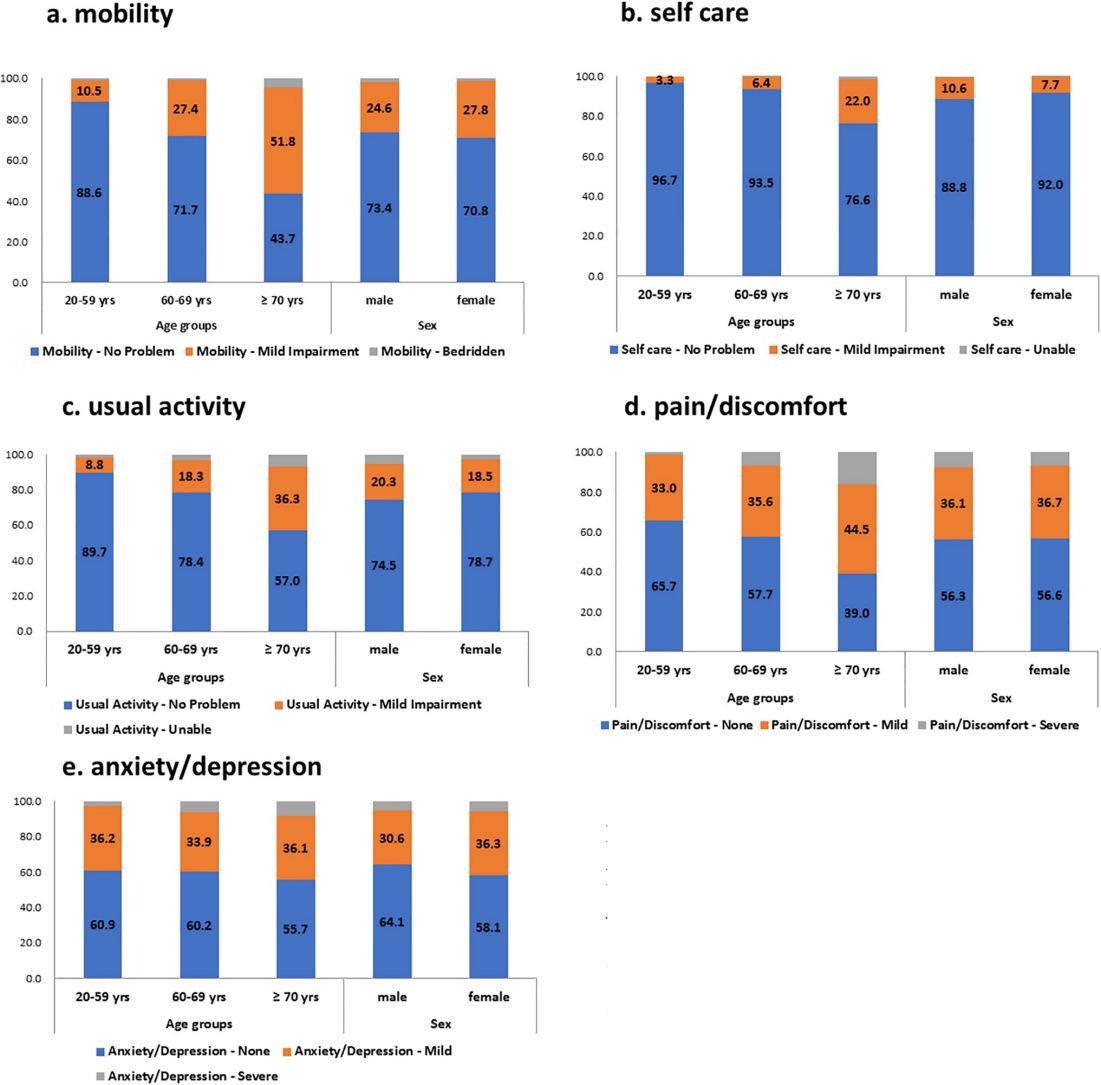
In accordance with gender and age groups, Proportions for each dimension of EQ-5D are visualized in a bar graph. All *p*-values were obtained by the Chi-Square Test and less than 0.05 except anxiety/depression category.

In Fig 1, the relations between age/sex and each dimension of EQ-5D are visualized in bar graphs. While sex showed insignificant associations with the EQ-5D dimensions, older age was correlated with problems in mobility, self-care, usual activities, and pain/discomfort. Anxiety/depression did not exhibit a statistically significant correlation with age.

Table 2 presents ORs for the EQ-5D dimensions, with sociodemographic characteristics as independent variables. Impaired mobility, higher age, lower level of education, low income, worse subjective perception of health, unemployment, and BMI ≥ 25 were linked to higher ORs. Problems in self-care were found with higher ORs in the oldest age group, lowest quartile of income, unemployment, and suicidal ideation. In terms of debilitations in usual activities, ORs showed an increase in higher age groups, lower education and income, worse self-perception of health, unemployment, and BMI ≥ 25. Pain/discomfort was linked to higher ORs in older age groups, worse self-perception of health, and BMI ≥ 25, and anxiety/depression to worse self-perception of health, melancholia, and suicidal ideation.

**Table 2:** ORs (95% CIs) for severe impairment in each EQ-5D dimension in accordance with socio-demographic and major clinical characteristics.

	Mobility	Self-Care	Usual Activity	Pain/ Discomfort	Anxiety/ Depression
Age group (≥70yr)	6.448(3.078,13.508)	11.03(2.68,45.391)	5.043(2.193,11.594)	2.561(1.437,4.564)	1.163(0.758,1.787)
Age group (60–69yr)	3.639(1.786,7.412)	3.554(0.911,13.871)	3.453(1.688,7.066)	1.656(1.021,2.686)	1.39(0.845,2.287)
Sex (male)		0.57(0.307,1.061)	0.63(0.404,0.982)		
Place (rural)	1.195(0.795,1.794)	0.654(0.359,1.189)	0.975(0.591,1.606)	0.995(0.656,1.509)	
Current smoker	0.699(0.404,1.207)				
Drinker	1.033(0.728,1.466)	1.15(0.641,2.064)	0.726(0.501,1.052)	0.986(0.716,1.358)	0.882(0.62,1.254)
Regular exercise	0.753(0.532,1.065)	0.695(0.41,1.178)	0.842(0.578,1.226)		
High education level	0.533(0.358,0.793)	0.742(0.387,1.419)	0.564(0.353,0.902)	0.716(0.506,1.014)	0.846(0.584,1.226)
Lowest income	2.061(1.383,3.071)	1.98(1.152,3.401)	2.79(1.918,4.057)	1.07(0.756,1.514)	1.451(0.981,2.146)
Spouse	0.686(0.46,1.024)	0.853(0.491,1.479)	0.598(0.399,0.895)	0.737(0.51,1.066)	0.839(0.583,1.206)
Self-perception of health (bad)	2.246(1.347,3.745)	2.236(0.782,6.394)	4.537(2.283,9.017)	5.257(3.228,8.56)	3.186(1.945,5.221)
Self-perception of health (usual)	0.96(0.555,1.661)	0.945(0.32,2.794)	1.107(0.529,2.315)	1.559(0.959,2.532)	1.476(0.88,2.475)
Non Employment	1.519(1.066,2.163)	1.981(1.041,3.771)	1.773(1.212,2.592)	1.345(0.967,1.871)	0.904(0.658,1.243)
Obesity	1.639(1.119,2.4)	1.617(0.902,2.899)	1.499(1.014,2.217)	1.661(1.152,2.394)	1.222(0.851,1.756)
Melancholia	1.223(0.834,1.794)	1.139(0.662,1.961)	1.296(0.885,1.898)	1.138(0.813,1.593)	2.676(1.902,3.765)
Suicidal ideation	1.477(0.985,2.215)	1.721(1.019,2.907)	1.309(0.896,1.913)	1.25(0.88,1.777)	1.789(1.283,2.497)
Metabolic syndrome	1.096(0.751,1.599)	0.63(0.365,1.085)	0.769(0.521,1.135)	0.742(0.523,1.055)	1.14(0.788,1.651)

Odd ratios (ORs) for the five EQ-5D dimensions were calculated by the multivariate logistic regression model, adjusting for age, sex, socio-demographic factors (education, household income, employment), and health-related factors (smoking, drinking, regular exercise, subjective perception of health, obesity and metabolic syndrome).

Table 3 shows the results of the analysis of covariance (ANCOVA) for EQ-5D, which adjusted for various factors examined in Table 1. Model 1 controlled for age and sex, and model 2 was adjusted for age/sex, sociodemographic factors (education, income, employment), and health-related factors (drinking, regular exercise, subjective perception of health, obesity, and metabolic syndrome). Various dimensions of mental health (stress, melancholia, suicidal ideation, professional consultation, and suicide attempts) showed statistically significant correlations with each EQ-5D item even after adjustment. This indicates that mental health problems can affect QoL by themselves, independent of other sociodemographic or health-related factors.

**Table 3:** The results of Analysis of Covariance (ANCOVA) of EQ-5D adjusted for age/sex, socio-demographic factors, and health-related factors.

	Model 1	Model 2
**Stress**		
No	0.882 ± 0.006	0.885 ± 0.006
Yes	0.806 ± 0.009	0.841 ± 0.007
*p*-value*	< 0.001	< 0.001
**Melancholia**		
No	0.884 ± 0.006	0.886 ± 0.005
Yes	0.798 ± 0.01	0.838 ± 0.007
*p*-value	< 0.001	< 0.001
**Suicide Idea**		
No	0.885 ± 0.005	0.887 ± 0.005
Yes	0.792 ± 0.01	0.833 ± 0.008
*p*-value	< 0.001	< 0.001
**Clinical Consult**		
No	0.855 ± 0.006	0.869 ± 0.005
Yes	0.816 ± 0.012	0.859 ± 0.009
*p*-value	0.002	0.3425
**Suicide Attempt**		
no	0.855 ± 0.005	0.872 ± 0.004
yes	0.725 ± 0.028	0.779 ± 0.027
*p*-value	< 0.001	< 0.001

* The *p*-values were obtained from Analysis of Covariance (ANCOVA).

Model 1 was adjusted for age and sex.

Model 2 was adjusted for age, sex, socio-demographic factors (education, household income, employment), and health-related factors (smoking, drinking, regular exercise, subjective perception of health, obesity and metabolic syndrome).

## Discussion

This KNHANES based study sought to identify risk factors that may lead to low QoL in people with depression. Among various factors examined, older age, lower level of education, lower income, unemployment, worse subjective perception of health, obesity and mental health struggles were associated with QoL impairments in depressive individuals after adjustment for multiple covariates.

Aging has adverse effects on QoL, especially for those enduring depression. Depression in later life is associated with increased risk of morbidity and mortality, and the depressed elderly are more likely to suffer from cognitive alterations, somatic symptoms, loss of interest, and inclinations to commit suicide [19]. Aging is a process of physical and biological change that inevitably entails numerous medical modifications that span neurological, cardiovascular, endocrine, inflammatory, and musculoskeletal changes. Elderly people may suffer from the multiple health disorders due to the vulnerability for many physical and mental disturbances.[20] Loneliness, functional disability or pain due to chronic disease, difficulties in vision or hearing and impaired sexual activity can decrease QoL of elderly.[21]

Results from our study have found lower socioeconomic status (lower income, lower education level, and unemployment) to be associated with low QoL. Chronic physical comorbidities and low socioeconomic conditions have been reported to have a negative impact on QoL.[22]. Similar to our findings, association between worse subjective perception and low QoL was confirmed in other studies as well.[23, 24] Obesity was also associated with lower QoL in our study. Several studies have shared similar results in reporting associations between obesity and decreased QoL.[25–28] The majority of published studies indicate that obesity impairs HRQoL, and that higher degrees of obesity are associated with greater impairment.[27]

Dimensions of mental health (stress, melancholia, consultation, suicidal ideation, and suicide attempt) were shown to impair QoL. In Table 3, it was confirmed that each of these dimensions retained significance after controlling for age/sex and for socioeconomic and health-related factors. According to the results, mental health seems to have an independent association with QoL. It has been reported that a significant burden of disease exists in MDD, and this burden increases as depression intensifies [29]. Studies have also reported improvements in subjective QoL after treatment of depression and amelioration of symptoms [30, 31].

In our study, smoking was not associated with QoL. Drinkers and patients who exercise regularly had higher QoL, but did not show any significant results after multivariate analysis. The association between HRQoL and drinking or smoking were not observed in several other studies as well.[32, 33] Therefore, it can be concluded that the effect of smoking and drinking on QoL of depressed patients is not significant.

The present study is not without limitations. First, as this study was based on a cross-sectional research design, it is difficult to establish causal relationships. Second, inclusion of melancholia (depression lasting more than two weeks) as a mental health-related factor and the anxiety/depression dimension of EQ-5D may act as a confounder in analyzing data drawn from a pool of depressive individuals. Third, this study did not exclude those who concurrently have other mental disorders, such as anxiety disorder, personality disorder, and schizophrenia. Further research may be directed towards confirming an association between EQ-5D levels and the severity of depressive symptoms. Other measures more specific to mental health, such as PHQ-9 (Patient Health Questionnaire-9), may be incorporated into future research. Advantages of this study are its large sample size and credible data source.

In conclusion, older age, lower level of education, lower income, unemployment, worse subjective perception of health, obesity and mental health struggles were associated with QoL impairments in depressive individuals. Patients with depression who have risk factors for low QoL should be identified and appropriately managed. This study is meaningful in that it provided grounding data for improving the QoL of depressed patients.
